# Genetic Engineering to Induce Fetal-Like Hematopoietic Stem Cells

**Published:** 2020

**Authors:** Bariş Ulum, Stefan A. Muljo

**Affiliations:** Integrative Immunobiology Section, Laboratory of Immune System Biology, National Institute of Allergy and Infectious Diseases (NIAID), National Institutes of Health (NIH), Bethesda, Maryland 20892, USA

**Keywords:** Regenerative Medicine, Precision Medicine, Cellular Engineering, Cellular Therapy, Prenatal Therapy, Hematopoietic Stem Cells, RNA-binding Proteins

## Abstract

Bone marrow transplantation (BMT) or hematopoietic stem cell transplantation (HSCT) is an archetype of cellular therapy. However, to date BMT still suffers from several complications. Recent technological advances have encouraged us to think about an alternative to traditional BMT. Specifically, we propose in utero HSCT (IUHSCT). For this purpose, we suggest that induced fetal-like hematopoietic stem cells (ifHSCs) might be suitable for IUHSCT, and should be seriously evaluated.

## DESCRIPTION

Bone marrow transplantation (BMT) or hematopoietic stem cell transplantation (HSCT) is an archetype of cellular therapy. Hematopoietic stem cells (HSCs) that reside in the bone marrow are responsible for regenerating hematopoiesis and the immune system upon BMT. Dr. E. Donnall Thomas was the first to perform BMT in a human patient [1,2], and consequently, won the Nobel Prize in Physiology or Medicine in 1990 for his pioneering work. BMT is recognized to cure many forms of human immunodeficiencies and hematological disorders. From 1957 to 2016, over 1.3 million BMTs have been performed worldwide [3]. However, to date BMT still suffers from several complications including but not limited to the following:

- **Pre-conditioning (myeloablation) prior to HSCT facilitates engraftment of transplanted HSCs in the bone marrow niche**. Usually, these regimens are very toxic, leave patients immunocompromised until the immune system is regenerated and can cause sterility and other developmental problems in pediatric patients [4,5]. Furthermore, there are some radiosensitive primary immunodeficiencies that have contraindications for DNA-damaging pre-conditioning regimens such as alkylating agents (eg. busulfan) or ionizing radiation [6]. Thus, would it not be ideal if there was an alternative procedure that did not require myeloablation?
- **Human leukocyte antigen (HLA) matching is critical** [7]. However, it can be challenging to find a genetically related or similar donor to supply histocompatible bone marrow-derived cells [8]. Thus, would it not be ideal if there was an alternative procedure that instead utilized a universal HSC?
- **Graft-versus-host disease (GVHD) is a common sequela**. It can cause lethality when the donor cells mount an immune reaction against the recipient patient’s tissues and organs [9]. Thus, would it not be ideal if there was an alternative procedure that did not have an incidence of GVHD?
- **Incomplete reconstitution following BMT is a problem that is not widely investigated.** When performed without myeloablation, Vely et al. reported that innate lymphoid cells are not regenerated [10]. Furthermore, we argue that BMT is not natural because principally it only reconstitutes the adult waves of hematopoiesis and completely bypasses the initial waves of development that occur prior to birth. Therefore, it is likely that other subsets of cells are not regenerated following this standard of care. For instance, in mice, a special subset of innate-like B cells called B-1 that are generated early in life are not reconstituted after BMT [11, 12]. Although this has not yet been investigated in humans, we predict that the same issue would occur in patients post-BMT. Thus, would it not be ideal if there was anxs alternative procedure that mimicked nature more closely?

**Figure 1:**
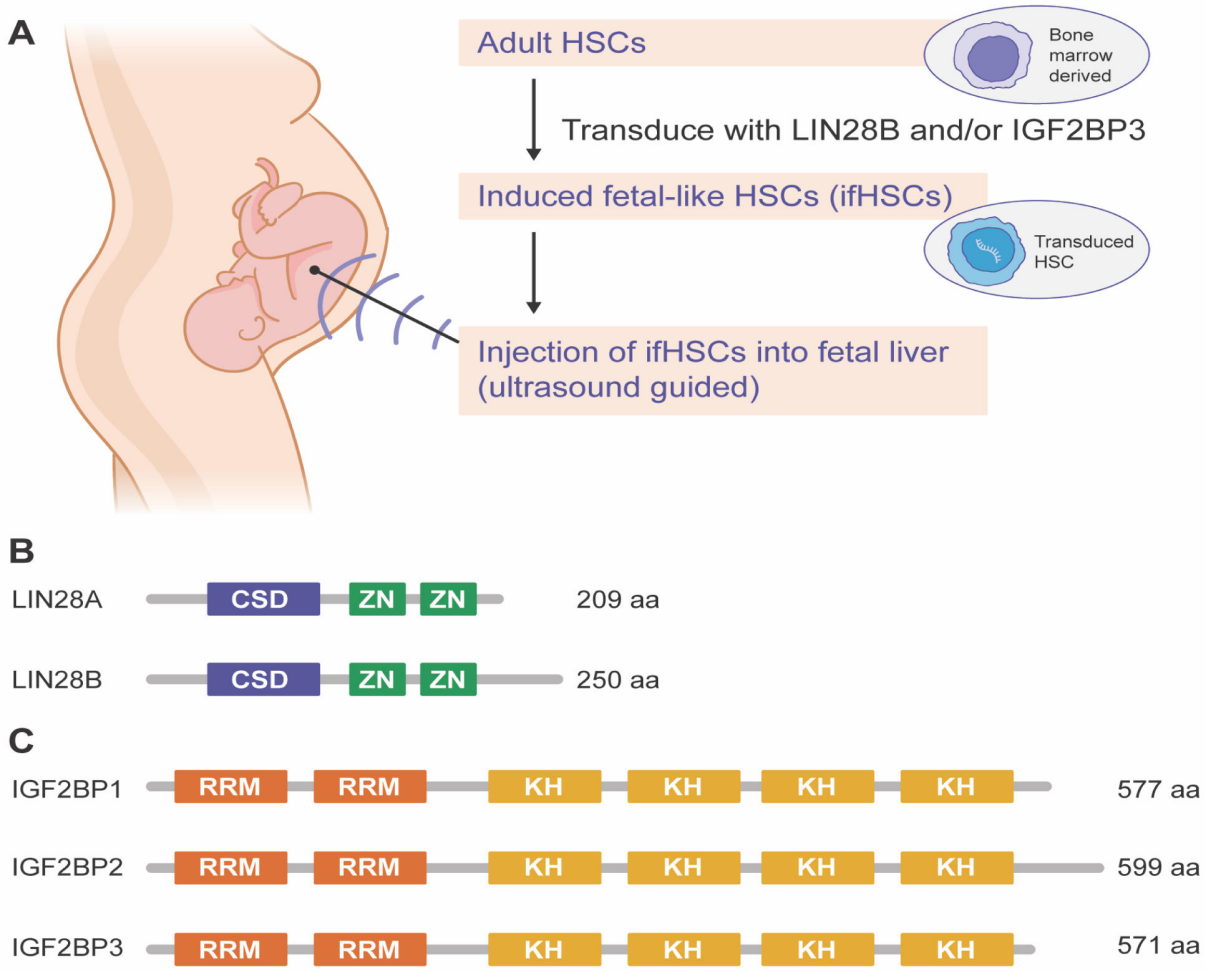
(A) A Regenerative Precision Medicine strategy aims to couple prenatal genetic diagnosis of inborn errors of hematopoiesis or immunity with IUHSCT. As an alternative to FL-derived HSCs, adult bone marrow-derived HSCs will undergo LIN28B and/or IGF2BP3-mediated reprogramming prior to IUHSCT. Reprogramming could be accomplished using approved viral vectors for gene therapy or by transfection of synthetic modified mRNAs encoding LIN28B and/or IGF2BP3. (B) Schematic depicts known domain structure of human LIN28A and LIN28B RNA-binding proteins. LIN28A is 209 amino acids (aa) long including the following domains: cold shock domain (CSD, aa 39 – 112), two CCHC-type Zinc (Zn) fingers (aa 137–154, and 159–176). LIN28B is 250 aa long including the following domains: CSD (aa 29–102), two CCHOtype Zn fingers (aa 127–144, and 149–166). Annotations are based on https://www.uniprot.org/uniprot/Q9H9Z2 and https://www.uniprot.org/uniprot/Q6ZN17 respectively. (C) Schematic depicts known domain structure of human IGF2BP1, IGF2BP2 and IGF2BP3 RNA-binding proteins. IGF2BP1 is 577 aa long including the following domains: two RNA-recognition motifs (RRM, aa 2–75, and 81–156), and four K homology domains (KH, aa 195–260, 276–343, 405–470, and 487–553). IGF2BP2 is 599 aa long including the following domains: two RRM (aa 3–76, and 82–157), and four KH domains (aa 193–258, 274–341, 427– 492, and 509–575). IGF2BP3 is 571 aa long including the following domains: two RRM (aa 2–75, and 81–156), and four KH domains (aa 195–260, 276–343, 405–470, and 487–553). Annotations are based on https://www.uniprot.org/uniprot/Q9NZI8, https://www.uniprot.org/uniprot/Q9Y6M1 and https://www.uniprot.org/uniprot/O00425 respectively.

These fundamental issues and recent technological advances have encouraged us to think about an alternative to traditional BMT. In the age of Precision Medicine, it is possible to diagnose many inborn errors of the hematopoietic or immune system prenatally [13-15]. Ideally, early diagnosis would be coupled with early treatment. It is not uncommon that such patients would need to wait years prior to receiving BMT, and in the meanwhile, they may suffer long term and/or life-threatening effects of their disease. Therefore, it is important to consider in utero HSC transplantation (IUHSCT, Figure 1A) for these cases [16]. Indeed, in sheep and monkeys, IUHSCTs have been demonstrated to work without the need for pre-conditioning, HLA matching or immunosuppression since GVHD was not observed [17, 18]. In these pioneering studies, investigators used HSCs derived from fetal liver (FL) as opposed to stem cells from adult donors which are commonly used for BMT.

In some countries, however, it is impossible to procure human FLs [19]. Research from our laboratory demonstrated that in principle it is possible to reprogram adult HSCs into a fetal-like state. In 2012, we identified the RNA-binding protein (RBP) Lin28b (Figure 1B) as one such factor capable of rejuvenating adult mouse HSCs and lymphoid progenitors [12]. Recently, we identified Igf2bp3 (Figure 1C), another RBP, as a second factor that determines the fetal hematopoietic fate [11]. Furthermore, we found evidence that Lin28b and Igf2bp3 cooperate to enact the fetal hematopoietic program [11]. Thus, we highlight the possibility of generating induced fetal-like HSCs (ifHSCs) which might be a suitable alternative to bona fide FL-derived HSCs (Figure 1A). Accordingly, we propose that ifHSCs should be seriously evaluated for IUHSCT in the future raising the hope that one day otherwise sick babies could be born cured.
